# Cancer stem cell immunoediting by IFNγ

**DOI:** 10.1038/s41419-023-06079-2

**Published:** 2023-08-21

**Authors:** Claudia Galassi, Lorenzo Galluzzi

**Affiliations:** 1 Department of Radiation Oncology, Weill Cornell Medical College, New York, NY USA; 2 Sandra and Edward Meyer Cancer Center, New York, NY USA; 3 Caryl and Israel Englander Institute for Precision Medicine, New York, NY USA

**Keywords:** Cancer stem cells, Immunosurveillance, Breast cancer

## Abstract

The secretion of interferon gamma (IFNG, best known as IFNγ) by immune effector cells generally mediates potent anticancer effects. Recent data from Beziaud et al. demonstrate that—at least in some circumstances—IFNγ can edit the breast cancer microenvironment to promote stemness, disease progression and resistance to (immuno)therapy.

Both natural and therapy-driven tumor-targeting immune responses generally involve the recognition of malignant cells by CD8^+^ cytotoxic T lymphocytes (CTLs) or natural killer (NK) cells, culminating with the release of effector molecules such as granzyme B (GZMB) and interferon gamma (IFNG, best known as IFNγ) [1]. Corroborating the key role of IFNγ in anticancer immunosurveillance, *Ifng*^*−/−*^ mice are more susceptible to carcinogen-driven tumorigenesis than their wild-type counterparts [2]. Moreover, mutations in the genes encoding interferon gamma receptor 1 (IFNGR1) or its signal transducer Janus kinase 2 (JAK2) have been associated with poor disease outcome in various cohorts of patients with cancer receiving immune checkpoint inhibitors (ICIs) [2]. That said, IFNγ signaling can also mediate pro-tumoral effects, for instance by promoting the upregulation of CD274 (best known as PD-L1), a co-inhibitory ligand with potent immunosuppressive effects [2, 3].

In multiple (preclinical and clinical) oncological settings, robust IFNγ responses emerge in the context of type I interferon (IFN) signaling, which not only promotes the direct activation of CD8^+^ CTLs and NK cells, but also engages additional immune effectors cells including dendritic cells (DCs) and T_H_1-polarized CD4^+^ T cells [4]. Similar to IFNγ, however, type I IFN can also exert pro-tumoral effects. For instance, suboptimal type I IFN signaling has recently been shown to promote the accumulation of cancer stem cells (CSCs) [5, 6], a poorly differentiated, immunoprivileged cancer cell subset that has been consistently associated with accelerated tumor progression and resistance to therapy [7]. Recent data from Beziaud et al. demonstrate that—at least in some circumstances—IFNγ can also promote cancer immunoediting toward increased stemness, rapid disease progression and (immuno)therapy resistance [8].

Beziaud et al. set to investigate whether peptide-based therapeutic vaccination would influence cancer stemness and metastatic disease dissemination in a mouse model of breast cancer expressing hormone receptors (HRs), at least at during early oncogenesis and tumor progression, as well as erb-b2 receptor tyrosine kinase 2 (ERBB2, best known as HER2). To this aim, they harnessed primary MMTV-PyMT cells to establish orthotopic mammary tumors in syngeneic immunocompetent C57BL/6J mice, followed by therapeutic vaccination with an antigenic epitope from Polyoma virus middle T (MT_245-253_), isolation of cancer cells forming progressing tumors (despite vaccination) and re-inoculation of such cells in tumor-naïve hosts. While vaccination mediated partial therapeutic effects in this model, MMTV-PyMT cells surviving vaccination exhibited superior metastatic potential upon intravenous re-inoculation in tumor-naïve hosts as compared to their counterparts from unvaccinated mice, a behavioral shift that was accompanied by increased expression of the CSC marker CD90 [8]. Pointing to an immunoediting mechanism in favor of stemness, CD90^−^ MMTV-PyMT cells exhibited increased sensitivity to CTL cytotoxicity as compared to their CD90^+^ counterparts. Moreover, MMTV-PyMT cells exposed in vitro to activated wild-type (but not *Ifng*^*−/−*^) T cells upregulated CD90, an effect that could be mimicked with recombinant IFNγ and could be abrogated by IFNγ neutralization or JAK1/2 inhibition with ruxolitinib [8]. These findings suggested that suboptimal tumor control by immune effector cells might promotes stemness via IFNγ.

Formally conforming this possibility, MMTV-PyMT cells exposed to recombinant IFNγ or culture medium conditioned by activated CTLs exhibited increased aldehyde dehydrogenase (ALDH) activity and sphere-forming capacity (two markers of bona fide CSCs) in an IFNγ-dependent manner. Moreover, IFNγ exposure not only increased the radio- and chemoresistance of MMTV-PyMT cells, knowing that CSCs are less sensitive to DNA damage than their normal counterparts [9], but also exacerbated their metastatic potential, irrespective of the immunological competence of the host. This latter finding was confirmed in a panel of human and mouse cancer cell lines with metastatic potential [8]. Altogether, these data demonstrate that—unless immunosurveillance results in tumor eradication—IFNγ signaling can promote the accumulation of aggressive CSCs underlying rapid tumor progression.

Next, Beziaud et al. harnessed RNA sequencing to identify the molecular players underlying the acquisition of stemness by MMTV-PyMT cells exposed to IFNγ, ultimately focusing on branched chain amino acid transaminase 1 (BCAT1)—an enzyme involved in branched chain amino acid degradation. Confirming this possibility, the BCAT1 inhibitor gabapentin (an FDA-approved agent for the treatment of epilepsy and neuropathic pain) efficiently prevented CD90 upregulation, increased sphere-forming potential and metastatic dissemination in IFNγ-exposed MMTV-PyMT cells. Similar results were obtained with in a panel of human and mouse cell lines. Similarly, while gabapentin only ameliorated the efficacy of therapeutic vaccination with MT_245-253_ to a marginal extent, persisting MMTV-PyMT cells had virtually null metastatic potential upon reinoculation in tumor-naïve hosts. The same held true when vaccination with MT_245-253_ was replaced with an ICI targeting programmed cell death 1 (PDCD1, best known as PD-1) [8]. Thus, suboptimal immunosurveillance as elicited by peptide vaccination or PD-1 blockage in insensitive tumors may result in the detrimental selection and expansion of pre-existing CSCs. Suggesting these findings may be relevant for patients with breast cancer, Beziaud et al. documented an increased frequency of CSCs in breast cancer patients 10 days after the initiation of an ICI targeting PD-1, as well as a positive correlation between CSC or BCAT1 signatures and IFNγ signatures, especially after treatment initiation [8].

In summary, Beziaud et al. delineated a new mechanism through which suboptimal anticancer immune responses as elicited by partially efficient (immuno)therapeutic regimens may select for aggressive cancer (stem) cell clones in support of rapid disease progression and therapeutic failure. Whether these findings are related to hyperprogression, i.e., the impressively rapid progression of a few patients with cancer treated with ICIs [10], remains to be formally assessed. Despite these and other unknowns, the recent data by Beziaud et al. raise the intriguing possibility that the detrimental effects of indolent type I IFN signaling on tumor progression and sensitivity to treatment [5, 6] may involve, at least in part, IFNγ-dependent immunoediting and the resulting selection of aggressive CSCs (Fig. 1). Additional work is needed to elucidate this possibility.

**Fig. 1:**
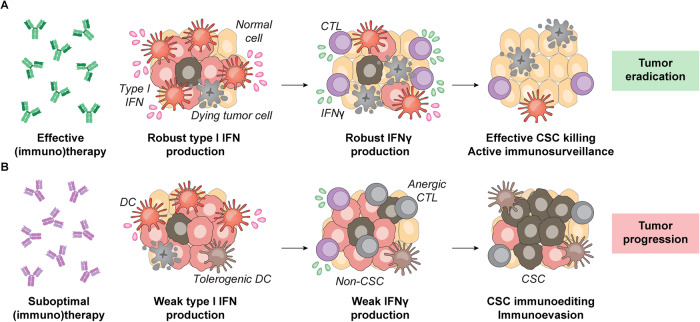
Detrimental effects of immunoediting on cancer stemness, tumor progression and resistance to therapy. Potent type I interferon (IFN) responses as driven by some (immuno)therapeutics appear to elicit robust CD8^+^ cytotoxic T lymphocyte (CTL) activity coupled with the abundant secretion of interferon gamma (IFNG, best known as IFNγ) and ultimately conducive to immunological tumor eradication (**A**). On the contrary, suboptimal type I IFN and/or IFNG signaling drive immunoediting in the tumor microenvironment (TME) in support of the selection of pre-existing—and/or generation of novel—cancer stem cells (CSCs), ultimately favoring accelerated disease progression in the context of (immuno)therapy resistance. At least in some settings, branched chain amino acid transaminase 1 (BCAT1) inhibition may limit CSC accumulation as driven by suboptimal type I IFN and/or IFNG signaling, thus restoring successful anticancer immunosurveillance (**B**). DC dendritic cell.
